# Histomorphometric and ultrastructural analysis of the tendon-bone interface after rotator cuff repair in a rat model

**DOI:** 10.1038/srep33800

**Published:** 2016-09-20

**Authors:** Tomonoshin Kanazawa, Masafumi Gotoh, Keisuke Ohta, Hirokazu Honda, Hiroki Ohzono, Hisao Shimokobe, Naoto Shiba, Kei-ichiro Nakamura

**Affiliations:** 1 Division of Microscopic and Development Anatomy, Department of Anatomy, Kurume University School of Medicine, Kurume, 830-0011, Japan; 2 Department of Orthopaedic surgery, Kurume University School of Medicine, Kurume, 830-0011, Japan

## Abstract

Successful rotator cuff repair requires biological anchoring of the repaired tendon to the bone. However, the histological structure of the repaired tendon-bone interface differs from that of a normal tendon insertion. We analysed differences between the normal tendon insertion and the repaired tendon-bone interface after surgery in the mechanical properties, histomorphometric analysis, and 3-dimensional ultrastructure of the cells using a rat rotator cuff repair model. Twenty-four adult Sprague-Dawley (SD) rats underwent complete cuff tear and subsequent repair of the supraspinatus tendon. The repaired tendon-bone interface was evaluated at 4, 8, and 12 weeks after surgery. At each time point, shoulders underwent micro-computed tomography scanning and biomechanical testing (N = 6), conventional histology and histomorphometric analysis (N = 6), and ultrastructural analysis with focused ion beam/scanning electron microscope (FIB/SEM) tomography (N = 4). We demonstrated that the cellular distribution between the repaired tendon and bone at 12 weeks after surgery bore similarities to the normal tendon insertion. However, the ultrastructure of the cells at any time point had a different morphology than those of the normal tendon insertion. These morphological differences affect the healing process, partly contributing to re-tearing at the repair site. These results may facilitate future studies of the regeneration of a normal tendon insertion.

A rotator cuff (RC) tear is a common soft tissue injury of the shoulder joint. Although surgical repair has become a primary treatment for RC tears, the failure rate of RC repair ranges from 20–70%, and most failures require surgical revision^1^,^2^,^3^,^4^. The repaired tendon-bone interface has been identified as a mechanical weak point, which may contribute to re-tearing^5^,^6^.

A normal tendon insertion attaches to the bone via fibrocartilage tissue, which consists of four zones: tendon, fibrocartilage, mineralised cartilage, and bone^7^,^8^. These structures functionally transfer the stress between dissimilar materials^9^. Transitions from mineralised cartilage to tendon are gradual and continuous, and there are no clearly-defined boundaries between the zones, even on an ultrastructural level^10^. Moreover, the transition zone, composed of mineralised cartilage and fibrocartilage, acts as a shock absorber by reducing the stiffness gradient between solid tissue (bone) and elastic tissue (tendon)^11^. These structures also contribute to the reduction in the tendon angulation that is necessary to prevent further shear stress^12^.

In contrast, repaired tendon anchors to the bone via fibro-vascular tissue; a fibrocartilaginous transition is rarely seen. The interface between the repaired tendon and bone has been found to be intermediated with disorganised scar tissue with type ІІІ collagen; these are gradually replaced with type І collagen without the type ІІ form^13^. Overall, this indicates a poor healing response to injury, with only partial recreation of the normal tendon insertion^14^.

However, many factors affecting the healing process at the tendon-bone interface remain unknown. Therefore, the differences between the repaired tendon-bone interface and a normal tendon insertion need to be clarified using innovative analysis.

In this study, we analysed the histomorphometric differences between the normal tendon insertion and the repaired tendon-bone interface after surgery, including the cellular distribution and 3-dimensional (3D) ultrastructure of the cells, using a rat RC repair model. Differences in the mechanical properties, amount of chondroid formation, and maturation of collagen bundles were also examined.

## Results

### Mechanical properties and bone mineral density

During the biomechanical testing, all specimens in the repaired group failed at the tendon-bone interface. In contrast, 5 of 6 specimens in the control group failed at the humeral head avulsion, while only one specimen failed at the supraspinatus tendon insertion site. The ultimate load-to-failure increased at each time point from 4 weeks to 12 weeks after surgery (4W: 17.2 ± 3.2 N, 8W: 26.3 ± 1.6 N, 12W: 36.5 ± 7.7 N). The linear stiffness (Normal: 61.5 ± 7.3 N/mm, 4W: 11.1 ± 2.0 N/mm, 8W: 21.5 ± 2.4 N/mm, 12W: 21.3 ± 5.2 N/mm) was significantly greater at the normal insertion compared to the repaired tendon-bone interface at all time points after surgery. Cross sectional area (Normal: 7.2 ± 0.5 mm^2^, 4W: 17.9 ± 4.5 mm^2^, 8W: 18.0 ± 2.0 mm^2^, 12W: 23.0 ± 2.4 mm^2^) was significantly smaller at the normal insertion compared to the repaired tendon-bone interface at any time point after surgery. Ultimate stress (Normal: 4.7 ± 1.7 MPa, 4W: 1.4 ± 0.5 MPa, 8W: 1.5 ± 0.1 MPa, 12W: 1.7 ± 0.5 MPa) and Young’s modulus (Normal: 20.0 ± 1.4 MPa, 4W: 2.1 ± 1.0 MPa, 8W: 3.0 ± 0.3 MPa, 12W: 3.8 ± 1.1 MPa) were significantly greater at the normal insertion compared to the repaired tendon-bone interface at any time point after surgery. Bone mineral density (BMD) increased significantly at each time point from 4 weeks to 12 weeks (4W: 648.6 ±23.1 mg/cm^3^, 8W: 740.6 ±2.7 mg/cm^3^, 12W: 774.2 ±8.9 mg/cm^3^) and was significantly greater at the normal insertion (672.8 ± 19.5 mg/cm^3^) compared to the repaired tendon-bone interface at any time point. These data are summarised in Table 1. The cross sectional area displayed a significant positive correlation with BMD (r = 0.62, p < 0.05) (Supplementary Figure S1). Other mechanical properties (ultimate load-to-failure, linear stiffness, ultimate stress, and Young’s modulus) were not significantly correlated with BMD.

### Histology

**Figure 1:**
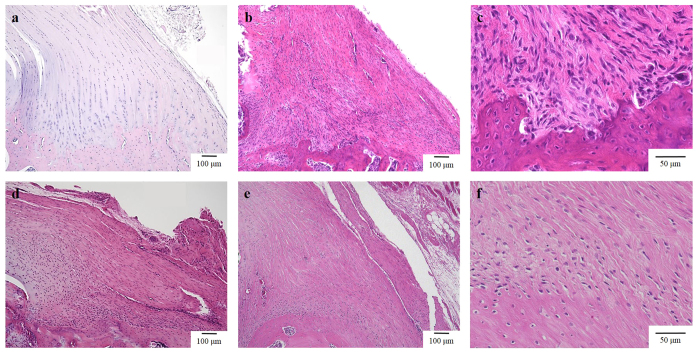
Histology of the tendon attachment site in a normal control specimen (**a)** ×100), and specimens from the rat rotator cuff repair model at 4 weeks (**b)** ×100) (**c)** ×400), 8 weeks (**d)** ×100), and 12 weeks (**e)** ×100) (**f)** ×400) post-surgery. At 12 weeks post-surgery, chondroid cells could be observed on the articular side. However, the entire shape of each cell could not be observed with light microscopy.

At the normal supraspinatus tendon insertion, each layer of the four zones (tendon, fibrocartilage, mineralised fibrocartilage, and bone) was observed. In particular, the boundaries between the mineralised fibrocartilage and the bone were evident. Although the boundaries between the fibrocartilage and mineralised fibrocartilage were not evident, the nuclei of the fibrocartilage could be distinguished from those of the mineralised fibrocartilage. Fibrocartilage and mineralised fibrocartilage were located in a columnar formation; simultaneously, matured collagen bundles were running between these cells (Fig. 1a). At 4 weeks after surgery, fibro-vascular tissue intervened between the repaired tendon and bone, and inflammatory cells were rarely seen between the repaired tendon and the bone. The cellular arrangement and collagen organization were immature compared to 8 and 12 weeks after surgery. Most of the nuclei of these cells were spindle-shaped (Fig. 1b). The perforating fibres between the tendon and bone were immature (Fig. 1c). At 8 weeks after surgery, a chondroid cell layer was observed between the repaired tendon and the bone. At this time point, the collagen organization and cellular arrangement had become more organized compared to 4 weeks. Additionally, perforating fibres to the bone were observed (Fig. 1d). At 12 weeks post-surgery, the collagen organization and cellular arrangement had become even more organized compared to 8 weeks (Fig. 1e); the number of perforating fibres also increased (Fig. 1f). In particular, chondroid cells could be observed on the articular side. However, the entire morphology of each cell could not be observed with conventional histology.

### Metachromasia

**Figure 2:**
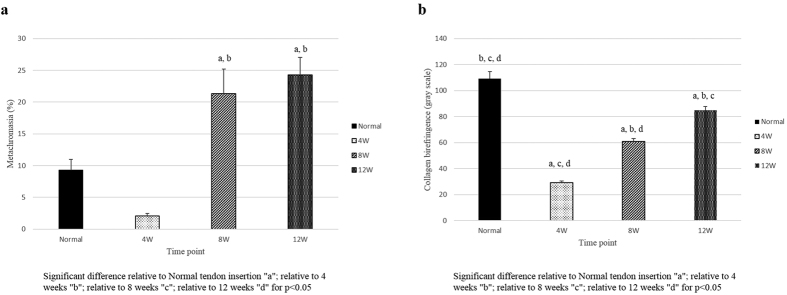
(**a**) Histogram showing metachromasia in the normal control and rotator cuff repair model at each time point post-surgery. Error bars represent the standard error. (**b**) Histogram showing collagen organization in the normal control and rotator cuff repair model at each time point post-surgery. Error bars represent the standard error.

The fraction of fibrocartilage area between the repaired tendon and the bone was analysed using safranin O staining. The fraction of fibrocartilage area was significantly greater at 8 and 12 weeks compared to 4 weeks. However, there were no significant differences between 8 and 12 weeks (4W: 2.1 ± 0.4%, 8W: 21.3 ± 3.8%, 12W: 24.3 ± 2.8%). The fraction of fibrocartilage area at the normal insertion (9.3 ± 1.7%) was greater compared to the repaired tendon-bone interface at 4 weeks after surgery; there were no significant differences between the normal insertion and the repaired tendon-bone interface at 8 or 12 weeks (Fig. 2a).

### Collagen organization

Collagen organization at the tendon attachment sites was analysed using picrosirius red staining. The collagen birefringence was as follows: 4W, 29.0 ± 1.4 grey scale units; 8W, 60.8 ± 2.4 grey scale units; and 12W, 84.8 ± 3.0 grey scale units. Birefringence increased significantly at each time point from 4 weeks to 12 weeks. However, it was significantly greater at the normal insertion (109. ± 5.5 grey scale units) compared to the repaired tendon-bone interface at any time point (Fig. 2b).

### Analysis of cellular distribution

**Figure 3:**
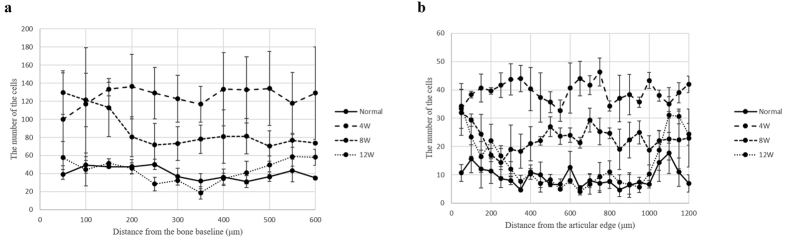
(**a**) Histogram showing the cellular distribution in the bone baseline area. Error bars represent the standard error. At 12 weeks post-surgery, there was no difference between the number of cells in the repaired tendon-bone interface and the normal insertion. (**b**) Histogram showing the cellular distribution in the tendon insertion area. Error bars represent the standard error. At 12 weeks post-surgery, the number of cells at the repaired tendon-bone interface was almost similar to that of the normal insertion.

Observation of the cellular distribution in the bone baseline area showed decreasing total cell numbers at each time point. As shown in Fig. 3a, at 4 weeks post-surgery, the number of cells (y-axis) increased until approximately 200 μm from the bone baseline (x-axis), and was maintained at approximately 130 cells every 50 μm for the remaining distances. However, at 8 weeks post-surgery, the number of cells decreased until a distance of approximately 200 μm and was maintained at approximately 80 cells every 50 μm after this distance. At 12 weeks post-surgery, there was no difference in the number of cells at each distance compared to that of the normal insertion. Similarly, observation of the cellular distribution in the tendon insertion area showed a decreasing number of cells at each time point. As depicted in Fig. 3b, at 4 weeks post-surgery the number of cells at each distance from the articular edge was maintained at approximately 40 cells every 50 μm. At 8 weeks post-surgery, the number decreased until 200 μm, but was maintained at approximately 20 cells every 50 μm beyond this distance. At 12 weeks post-surgery, the number of cells at each distance was similar to that of the normal insertion, and the cell number varied with distance more than it did at 4 and 8 weeks. The number of cells at both sides of the tendon edge was greater than the number in the central portion of the insertion.

### Ultrastructural analysis using focused ion beam/scanning electron microscope (FIB/SEM) tomography

**Figure 4:**
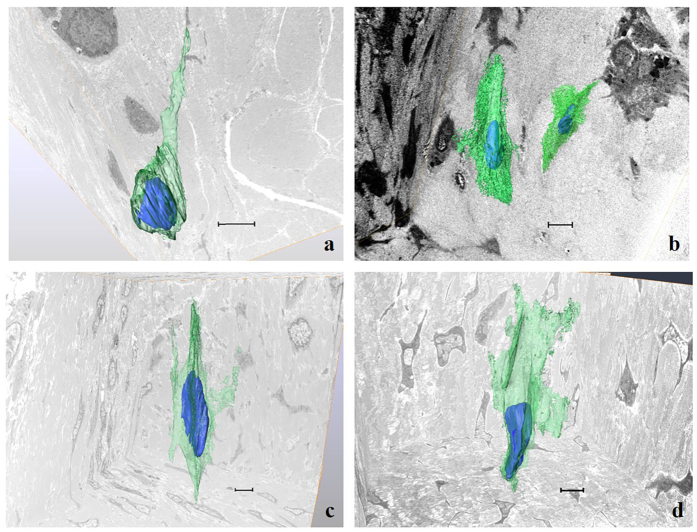
3D reconstructed images of the cells at the tendon attachment site of the normal control (**a**), at 4 weeks (**b**), 8 weeks (**c**), and 12 weeks (**d**) post-surgery. Scale bar represents 5 μm. The cellular ultrastructure of the tendon-bone interface at all time points after rotator cuff repair is different from that of the normal tendon insertion. (blue: cell nucleus; green: cell cytoplasm).

In the control specimens, most of the cells at the supraspinatus tendon insertion were chondroid-shaped. These cells were located between mature collagen bundles and arranged with their cell processes parallel to the bundles. 3D-reconstructed images showed that the processes of these cells were oriented in the same direction as the adjacent collagen bundles. No cell-to-cell connections via the processes were observed (Fig. 4a). At 4 weeks after surgery, apparent boundaries separating the fibro-vascular tissue from the bone were observed. Most of these cells were ellipsoidal in shape. However, some of the cells passed their processes over the interface between the fibro-vascular tissue and bone (Fig. 4b). At 8 weeks after surgery, the cells at the tendon attachment site were located between the mature collagen bundles. Most of the cells were ellipsoidal in shape and irregularly extended their cell processes, contacting each other (Fig. 4c). At 12 weeks after surgery, most of the cells were ellipsoidal, and their cellular processes were more regularly extended in a consistent direction compared to the cells at 4 and 8 weeks. In addition, the cellular processes were broader and flattened compared to those at 4 weeks, 8 weeks, and the normal insertion (Fig. 4d).

## Discussion

The present study investigated the initial healing process at the tendon-bone interface following RC repair by integrating various evaluation techniques. We demonstrated that the cellular distribution at the repaired insertion site becomes similar to the normal tendon insertion at 12 weeks after surgery, although the ultrastructure of the cells is quite different between the repaired and control groups at all time points after surgery.

First, we examined the repaired tendon-bone interface using conventional methods (mechanical properties, H&E, safranin O, and picrosirius red staining). These analyses demonstrated that the ultimate load-to-failure increased with time post-surgery in a manner that was dependent on the cross sectional area. The ultimate load-to-failure was equivalent between the control and repaired specimens at 8 and 12 weeks, though most control specimens failed at the humeral head rather than the tendon insertion. In contrast, linear stiffness, ultimate stress, and Young’s modulus did not reach the values of the normal tendon insertion by 12 weeks post-surgery. Thus, post-surgical mechanical properties could not fully recover to those of a normal tendon insertion, even after 12 weeks. These findings are consistent with previous studies^15^,^16^,^17^,^18^,^19^. In addition, the formation of fibrocartilage (data from safranin O staining) and the collagen organization (data from picrosirius red staining) at 12 weeks after surgery were not equivalent to the normal tendon insertion. The formation of fibrocartilage and collagen organization in this study are similar to the results of previous reports of repaired tendon-bone interfaces up to 8 weeks after surgery^16^,^19^,^20^,^21^,^22^,^23^.

Since cellular distribution is an important factor for re-establishing a normal tendon insertion, we examined the cellularity at the repaired tendon-bone interface. Our data showed that the number of cells between the repaired tendon and bone at 4 weeks after surgery initially increased; at 12 weeks after surgery, this number decreased to that of the normal tendon insertion. The number of the cells close to the frontline of the mineralization was larger than the number of cells in the tendon substance. In addition, the number of cells on both sides of the intervening tissue was larger than the number in the mid-portion area. Eventually, the cellular distribution at 12 weeks post-surgery almost mirrored that of the normal tendon insertion; the cells were located in a columnar formation.

Numerous histological analyses of tendon-to-bone healing have been performed in previous studies^6^,^13^,^20^,^21^,^24^,^25^,^26^,^27^,^28^,^29^,^30^,^31^,^32^,^33^,^34^,^35^. However, it was impossible to analyse the 3D ultrastructure of the cells between the tendon and bone after surgery at the transmission electron microscopic level. The advent of FIB/SEM tomography is revolutionary in its ability to analyse the 3D ultrastructure of cells with the high resolution of a transmission electron microscope^10^,^36^,^37^,^38^,^39^,^40^. Utilizing this method, we successfully determined the 3D ultrastructure of the cells between the repaired tendon and bone, and then correlated the whole ultrastructure of the cells with collagen bundles.

In the 3D ultrastructure, most of the cells at the normal tendon insertion were chondroid-shaped and had a few cell processes, which were oriented in the same direction as the adjacent collagen bundles, as previously reported^10^. These cells were also located between mature collagen bundles, and their cell processes were spindle-shaped. In contrast, after RC repair, the cells became ellipsoidal in shape; they possessed many cell processes, which were irregularly extended and in contact with each other. These cell processes were also broad and flat. As a result, even at 12 weeks after surgery, most of the cells between the repaired tendon and bone were different from the cells at the normal tendon insertion. Previously, we described the development of the normal tendon insertion using FIB/SEM tomography^40^ and revealed that the morphology of the cells was drastically transformed at 4 weeks post birth. Comparing the tendon-to-bone healing after surgery with the development of the normal tendon insertion, the process of transformation of the cells at the tendon attachment site is also different. These results strongly suggest that these morphological differences greatly affect the healing process, partly contributing to re-tearing at the repaired site.

In the present study, the cross sectional area and BMD were positively correlated. Previous studies have shown that the application of various factors for bone regeneration could affect the ultimate load-to-failure between the repaired tendon and bone^41^,^42^,^43^,^44^ our data are consistent with these reports. These phenomena indicate that the mineralisation activity on the bone side could influence the production of the intervening tissue between the repaired tendon and bone. On the contrary, other factors (ultimate load-to-failure, ultimate stress, linear stiffness, and Young’s modulus) were not significantly affected by BMD, indicating that activity at the bone side did not affect the maturation of the intervening tissue (in particular, organization of the collagen bundles) between the repaired tendon and bone. The influence of the biological environment at the bone on the regeneration of the repaired insertion remains to be clarified.

Although the presence of perforating fibres between repaired tendons and bones has been reported, the production process of these fibres has not been clarified^6^,^9^,^13^. In the present study, perforating fibres were seen at 4 weeks after surgery using FIB/SEM tomography, and we detected cells passing over the interface between the fibro-vascular tissue and the bone at the repaired site, possibly associated with the establishment of the perforating fibres. The perforating fibres produced by these cells could play a crucial role in the regeneration of the tendon-bone interface.

This study has several limitations. First, the findings obtained from these data may differ from those involving human subjects. Although rat RC repair has been utilised as a standard model to evaluate tendon-to-bone healing following RC repair, and previous reports have shown that the rat shoulder closely approximates the human shoulder in terms of anatomy^45^, we must keep these differences in mind before applying these data clinically. Second, the sample size was limited. The mechanical analysis largely served to investigate the validity of the study design. We determined that the sample size was appropriate based on the statistically significant differences in the mechanical properties. In the histomorphometric analyses, including metachromasia, collagen organization, and cellular distribution, we present these results with an emphasis on the histological tendencies rather than statistical differences. We believe that these histomorphometric data are pertinent for clarifying tendon-to-bone healing. Further studies with larger sample sizes are required to quantitatively verify the histological changes involving the repaired tendon-bone interface. Third, we used qualitative rather than quantitative analysis for the 3D ultrastructural analyses. Although FIB/SEM tomography has a higher resolution than conventional histology with light microscopy, the area of acquisition with FIB/SEM tomography was small to analyse quantitatively. Thus, further studies are necessary to perform a quantitative analysis with the resolution of an electron microscope. Finally, this study is a comparative study of the initial tendon-to-bone healing following RC repair using a rat model. Therefore, we did not utilize any applications to accelerate or regenerate the tendon-bone interface. However, we believe our findings benefit future studies that intend to evaluate tendon-to-bone healing utilising various applications.

In summary, our study describes the initial healing process between the repaired tendon and bone after RC surgical repair. The cellular distribution between the repaired tendon and bone at 12 weeks was similar to that of a normal tendon insertion. However, the 3D ultrastructure of the cells was completely different from that of the normal tendon insertion. This information may facilitate upcoming studies on the regeneration of a normal tendon insertion. Further studies are necessary to clarify the natural healing process between repaired tendons and bones, in order to regenerate a normal tendon insertion between these structures.

## Materials and Methods

### Study Design

**Figure 5:**
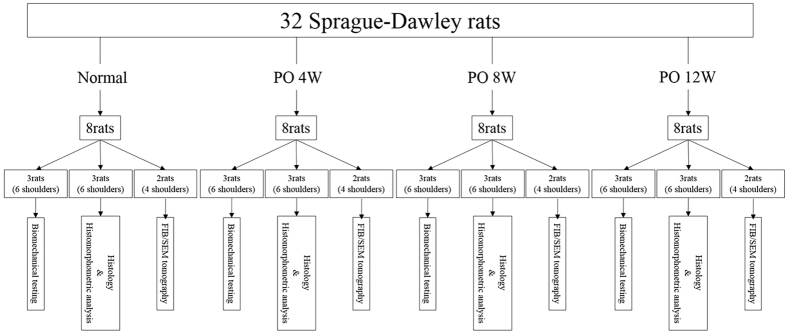
Flow diagram of the study design, illustrating how the rats were divided into groups for the three time points and the control group.

All experiments were performed in accordance with the National Institutes of Health Guidelines for animal research. This study was approved by the ethics review board of the Kurume University Animal Care Centre. Thirty-two adult Sprague-Dawley (SD) rats (mean body weight, 429.4 ± 72.9 g) were used in this study: 24 rats underwent complete cuff tear and subsequent repair of the supraspinatus tendon, and 8 served as normal controls. The repaired supraspinatus tendon-bone interface was evaluated at 4, 8, and 12 weeks after surgery. At each time point, 6 shoulders underwent micro-computed tomography (CT) scanning and biomechanical testing to evaluate their bone mineral density (BMD) and mechanical properties. Six shoulders were subjected to conventional histology and histomorphometric analysis using haematoxylin and eosin (H&E), safranin O, and picrosirius red staining. The remaining 4 shoulders underwent ultrastructural analysis using FIB/SEM tomography. The supraspinatus tendon insertions of 8 age-matched adult SD rats were used as normal controls (Fig. 5).

### RC repair model

**Figure 6:**
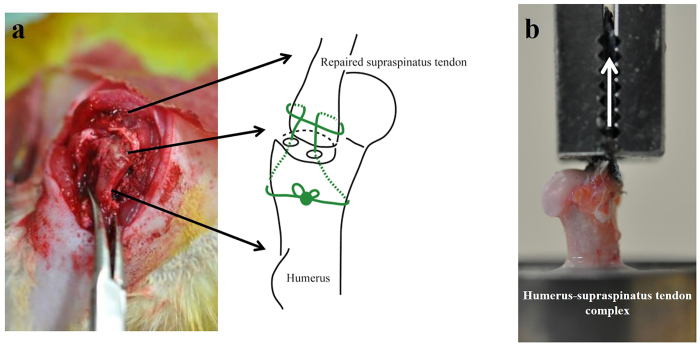
(**a**) Photographs and scheme showing the supraspinatus tendon repair conducted on a rat shoulder. (**b**) Photographs showing the mechanical testing. The repaired tendon-humerus complex was positioned to allow tensile loading in the longitudinal direction of the repaired tendon-humerus interface.

Each rat was anaesthetized with isoflurane under high flow oxygen, and supraspinatus tendon repair was subsequently performed. A midline skin incision was made and the subcutaneous tissues were divided. After the deltoid was divided to expose the shoulder joint, the supraspinatus tendon insertion was excised at the bone attachment site with a #11 scalpel blade. A suture was passed through the free end of the supraspinatus tendon using the Krackow technique. Using a 0.5-mm drill bit, the remaining normal tendon insertions were debrided. Two small bone tunnels for pull-out were created from the bone attachment site to the greater tuberosity; the sutures were pulled out to the bone tunnels and firmly secured to the lateral cortex under suitable pressure. The wound was then closed in layers. The animals were allowed to move freely in their cages after the operation (Fig. 6a).

### Bone mineral density using micro-computed tomography

The BMD of the supraspinatus tendon attachment site in the humerus was assessed using micro-CT (R-mCT2, Rigaku Corporation, Japan); at each time point, 6 specimens underwent micro-CT before biomechanical testing. All specimens were tested immediately after sacrifice. Soft tissues over the humerus were removed except for the repaired supraspinatus tendon-humerus complex. The sutures that had been used to fix the repaired tendon to the bone were cut before testing. The repaired tendon was secured in a screw grip using sandpaper and ethyl cyanoacrylate. Micro-CT was performed on the day of sacrifice, with the specimens in saline. Each sample was placed in the holder and scanned at 90 kV and 160 μA. Phantom images of a bone reference material were also acquired for calibration. The acquired images were analysed with BMD analysis software (TRI/3D-BON, Ratoc System Engineering CO, Japan). The images were thresholded 5 mm from the proximal end of the humerus head (Supplementary Figure S2a). The bone volume (BV) is the total number of thresholded bone voxels within the total volume (TV) of the volume of interest (VOI). After thresholding, the total bone mineral content, BV fraction (BV/TV), and BMD were calculated for the VOI at the supraspinatus tendon attachment site. For analysis of the bone mineral, the threshold was set to 350 Hounsfield units. Each specimen immediately underwent biomechanical testing after scanning.

### Biomechanical testing

Following micro-CT scanning, the specimens were placed into a tensile testing machine (TENSILON RTE-1210; Orientec) (Fig. 6b). The humerus was secured in a custom-designed pod using a capping compound^46^,^47^. The repaired tendon-humerus complex was positioned to allow tensile loading in the longitudinal direction of the repaired tendon-humerus interface. The specimens were preloaded to 0.1 N for 5 minutes, followed by 5 cycles of loading and unloading at a cross head speed of 5 mm/min, and then loaded to failure at a rate of 1 mm/min; the mechanical properties were then calculated. Failure modes were recorded for each specimen. Ultimate load-to-failure was recorded as the peak load before failure. Linear stiffness was calculated by determining the slope of the linear portion of the load-elongation curve. Ultimate stress was calculated by dividing the ultimate load-to-failure by the cross sectional area of the repaired tendon-bone interface, obtained from the axial section of the micro-CT image (Supplementary Figure S2b). Young’s modulus was calculated by determining the slope of the linear portion of the stress-strain curve. The strain was calculated by dividing the elongation by the initial length obtained from the coronal section of the micro-CT image. This testing protocol was similar to that described previously^20^,^21^.

### Conventional histology and histomorphometric analysis

At each time point, the specimens were fixed in 10% buffered formalin and then decalcified with Kalkitox solution (Wako Pure Chemical Industries, Ltd., Osaka, Japan). Tissues were processed for paraffin embedding. Longitudinal sections with a thickness of 5 μm at the centreline of the repaired tendon-bone interface were placed on glass slides and stained with H&E, safranin O, and picrosirius red. The specimens were visualized under a light microscope (BZ-X710; Keyence, Osaka, Japan) and a polarized light microscope (OLYMPUS BX50; OLYMPUS, Tokyo, Japan), and photomicrographs were obtained.

### Metachromasia

The fibrocartilage areas at the tendon attachment sites were analysed using safranin O staining. Six slides for each time point were randomly selected, and the images were obtained at 4 × magnification. The fibrocartilage areas were carefully outlined and measured using ImageJ software (NIH, Bethesda, MD). All images were analysed in a blinded fashion by two independent investigators. For the normal control samples, a total of 4 photomicrographs were taken and analysed, given the limited sample size.

### Collagen organization

Collagen organization at the tendon attachment sites was analysed using picrosirius red staining^15^,^16^,^21^,^22^,^48^. At each time point, 5 glass slides were randomly selected for semi-quantitative analysis; 3 photomicrographs were taken at 100× magnification for each slide. A total of 45 photomicrographs (15 photomicrographs at each time point) at the tendon attachment site after surgery were digitized (8-bit) using ImageJ software at a resolution of 1360 × 1024 pixels. For the normal control group, 5 photomicrographs were taken and analysed, because the normal supraspinatus tendon insertions were much smaller compared to the repaired tendon-bone interfaces after surgery.

### Analysis of cellular distribution

For cellular distribution analysis, 3 glass slides were randomly selected from each time point, and photomicrographs were taken at 10× magnification. The whole image of the normal tendon insertion or repaired tendon-bone interface was trimmed to 2 areas: 1) bone baseline area, and 2) tendon insertion area. To determine the bone baseline area, the boundary between the bone and mineralised cartilage layer was manually plotted, and a fitted line (bone baseline) was made. Utilising this as the bottom line, a square image of 1000 × 1000 pixels was trimmed. To determine the tendon insertion area, the articular edge and bone baseline were plotted, and a fitted line was similarly made. By employing these lines as the width and height, a rectangular image of 1920 × 266 pixels was trimmed. Following this, the nuclei of the cells were manually thresholded, and the centroid of each cell was calculated (Supplementary Figure S3). All calculated data were sorted by their distance from the base line at every 50 μm (1: from bone baseline, 2: from articular edge); a histogram was created for the number of cells (y-axis) versus the distance from the base line (x-axis), and each time point was compared in terms of this cellular distribution (Fig. 3a,b).

### Ultrastructural analysis using FIB/SEM tomography

The ultrastructure of the cells at each time point was analysed using FIB/SEM tomography, a new scanning electron microscopy method^10^,^36^,^37^,^38^,^39^,^40^. For each time point, rats were anaesthetised with diethyl ether and sodium pentobarbital, which was transcardially perfused through the left ventricle with heparin-containing saline, and then fixed in half Karnovsky solution (2% paraformaldehyde, 2.5% glutaraldehyde, 2 mM CaCl_2_ in a 0.1 M cacodylate buffer). After perfusion, the repaired tendon-humerus complexes were further immersed in the same fixative. After decalcification, the specimens were cut and further fixed with ferrocyanate and 1% osmium tetroxide (OsO_4_). Subsequently, the specimens were treated with 1% thiocarbohydrazide and were then immersed in a 1% OsO_4_ solution. For en bloc staining, the specimens were immersed in a solution of 4% uranyl acetate solution overnight and washed with distilled water. The specimens were further stained with Walton’s lead aspartate solution and dehydrated with a graded ethanol series, infiltrated with an epoxy resin mixture, and polymerised at 60 °C for 72 h. The surface of each embedded specimen was exposed with a diamond knife, and the resin blocks were trimmed and placed on an appropriate holder. Each specimen was fixed to the stage of the FIB/SEM machinery (Quanta 3DFEG; FEI, Hillsboro, OR, USA). Serial images of the block face were acquired by repeated cycles of sample surface milling using a focused gallium ion beam (milling step: 100 nm, 700 cycles) and by image acquisition using SEM as a compositional contrast image from secondary electrons (landing energy, 2.5 keV). The reconstructed images covered the supraspinatus tendon insertion or repaired tendon-attachment site to the bone. Approximately 700 block face images could be obtained per specimen. The morphology of the cells was evaluated after 3D reconstruction using Amira 5.5 software (FEI). The 3D morphology of the cells was extracted using a semi-manual procedure^10^,^39^,^40^. Briefly, all image stacks were normalised using a histogram-based image filter, and a median filter was applied to prepare for threshold segmentation. The cellular regions were selected using the threshold method; irregular regions in the selected regions were manually removed.

### Statistical analysis

Statistical analysis was performed using JMP version 11 (SAS Institute INC., Cary, NC). Data were expressed as the mean and standard error. The Kruskal-Wallis and Wilcoxon tests were used to evaluate the normal tendon insertion and repaired tendon-bone interface at each time point by comparing their BMD, mechanical properties, and histomorphometric values. The Spearman correlation rank test was used to evaluate the relationship between the mechanical properties and BMD. Differences with p < 0.05 were considered significant.

## Additional Information

**How to cite this article**: Kanazawa, T. *et al.* Histomorphometric and ultrastructural analysis of the tendon-bone interface after rotator cuff repair in a rat model. *Sci. Rep.* **6**, 33800; doi: 10.1038/srep33800 (2016).

## Supplementary Material

Supplementary Information

## Figures and Tables

**Table 1:** Mechanical properties and bone mineral density.

	Ultimate load-to-failure (N)	Ultimate stress (Mpa)	Linear stiffness (N/mm)	Cross Sectional Area (mm^2^)	Young’s modulus (Mpa)	Bone Mineral Density (mg/cm^3^)
Normal tendon insertion	34.6 ± 3.5^b^	4.7 ± 0.7^b^,c,d	61.5 ± 7.3^b^,c,d	7.2 ± 0.5	20.0 ± 1.4^b^,c,d	672.8 ± 19.5
4 weeks	17.2 ± 3.2	1.4 ± 0.5	11.1 ± 2.0	17.9 ± 4.5^a^	2.1 ± 1.0	648.6 ± 23.1
8 weeks	26.3 ± 1.6^b^	1.5 ± 0.1	21.5 ± 2.4^b^	18.0 ± 2.0^a^	3.0 ± 0.3	740.6 ± 2.7^a^,b
12 weeks	36.5 ± 7.7	1.7 ± 0.5	21.3 ± 5.2	23.0 ± 2.4^a^	3.8 ± 1.1	774.2 ± 8.9^a^,b,c

All values are mean ± standard error. Significant difference relative to

^a^Normal tendon insertion and

^b^4 weeks.

^c^8 weeks, and

^d^12 weeks after rotator cuff repair; p < 0.05.
