# Supplementary material for: Histomorphometric and ultrastructural analysis of the tendon-bone interface after rotator cuff repair in a rat model
Source: Sci Rep. 2016 Sep 20;6:33800. doi: 10.1038/srep33800 (PMC5028779; doi:10.1038/srep33800)
Supplement: Supplementary Information [file srep33800-s1.pdf]

Histomorphometric and ultrastructural analysis of the tendon-bone interface after rotator cuff repair in a rat model

Tomonoshin Kanazawa[1,2*], Masafumi Gotoh[2], Keisuke Ohta[1], Hirokazu Honda[2], Hiroki Ohzono[1], Hisao Shimokobe[2], Naoto Shiba[2], and Kei-ichiro Nakamura[2]

[1]Division of Microscopic and Development Anatomy, Department of Anatomy, Kurume University School of Medicine, Kurume, 830-0011, Japan

[2]Department of Orthopaedic surgery, Kurume University School of Medicine, Kurume, 830-0011, Japan

*Corresponding author; Tomonoshin Kanazawa

Email; tomochan@med.kurume-u.ac.jp

Telephone number; +81-942-31-7541

Fax number; +81-942-31-7555

Supplementary, Figure S1

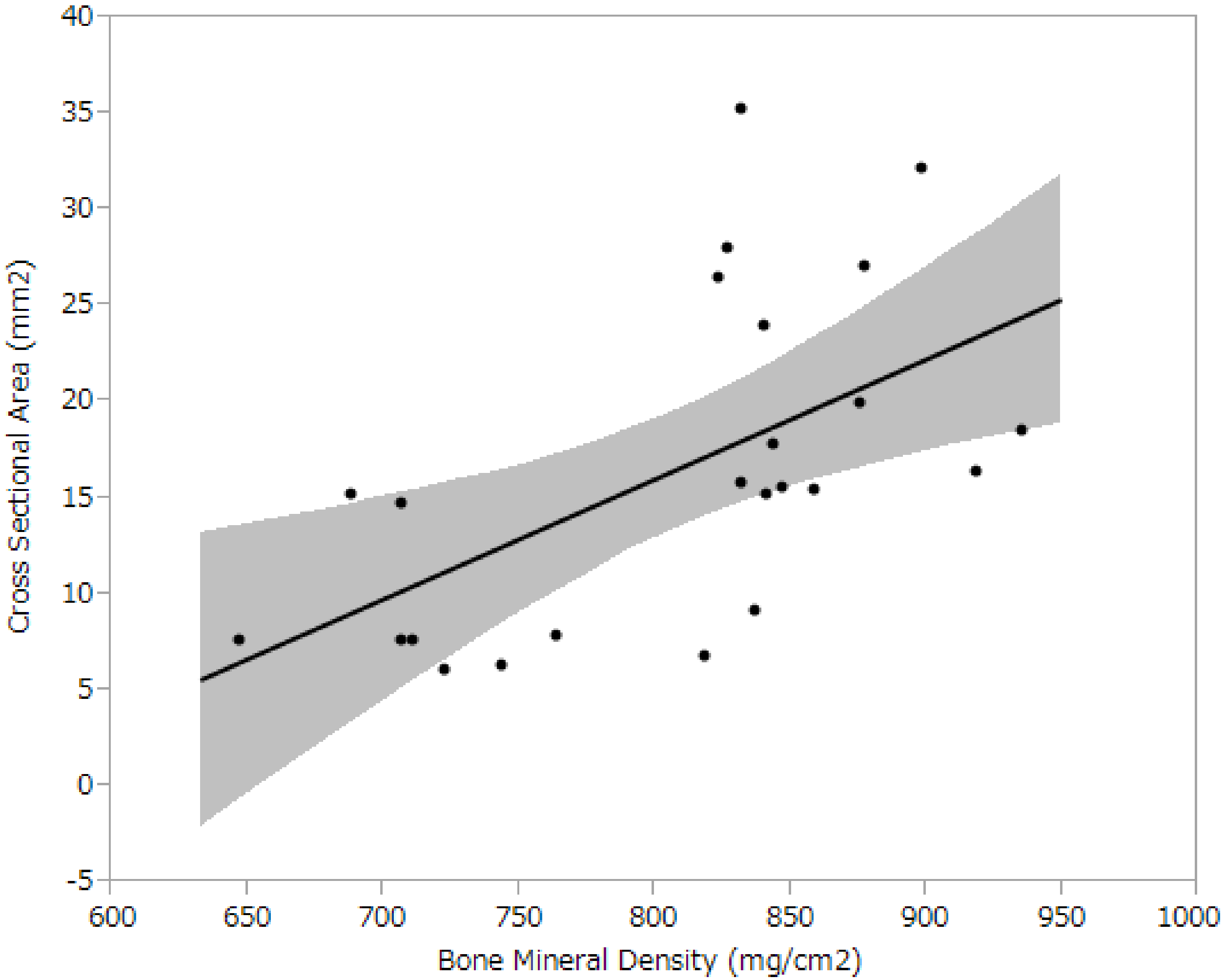

Supplementary, Figure S2

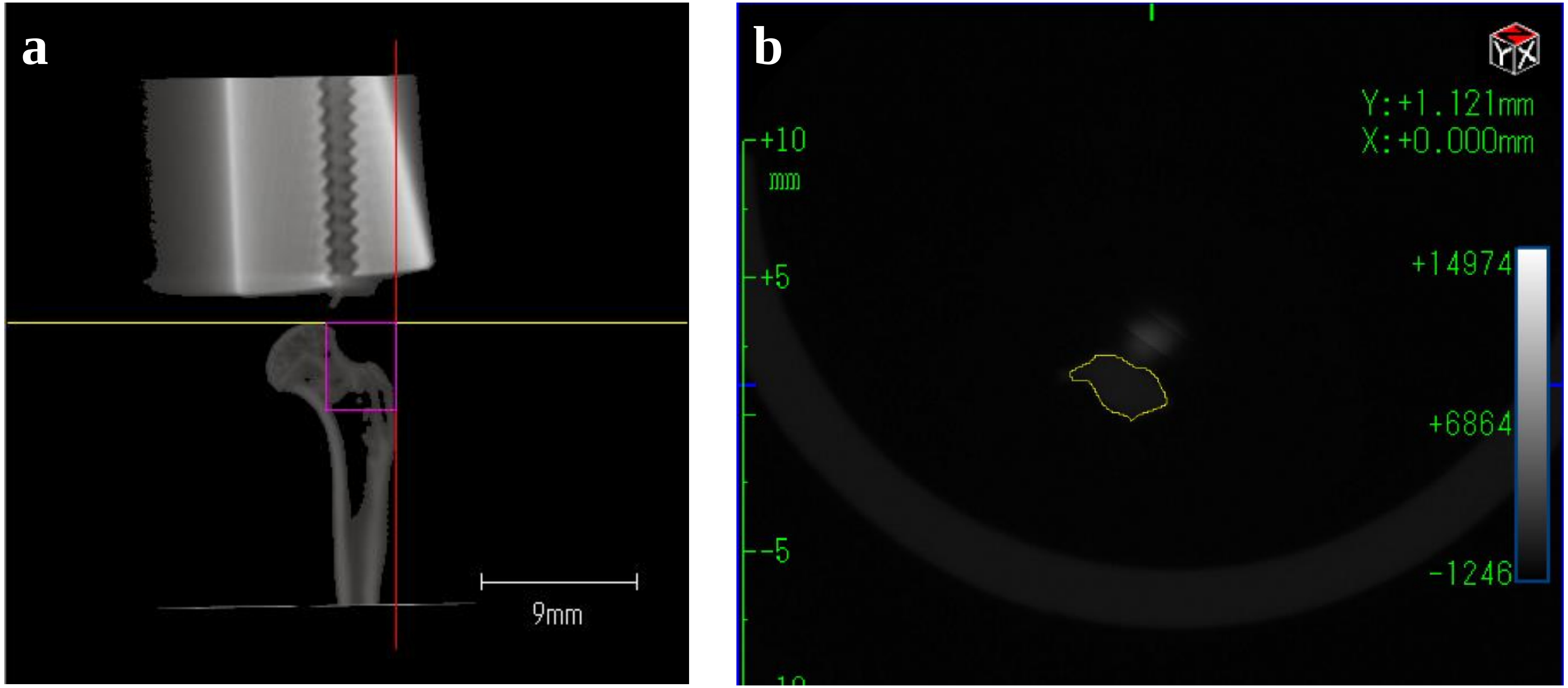

Supplementary, Figure S3

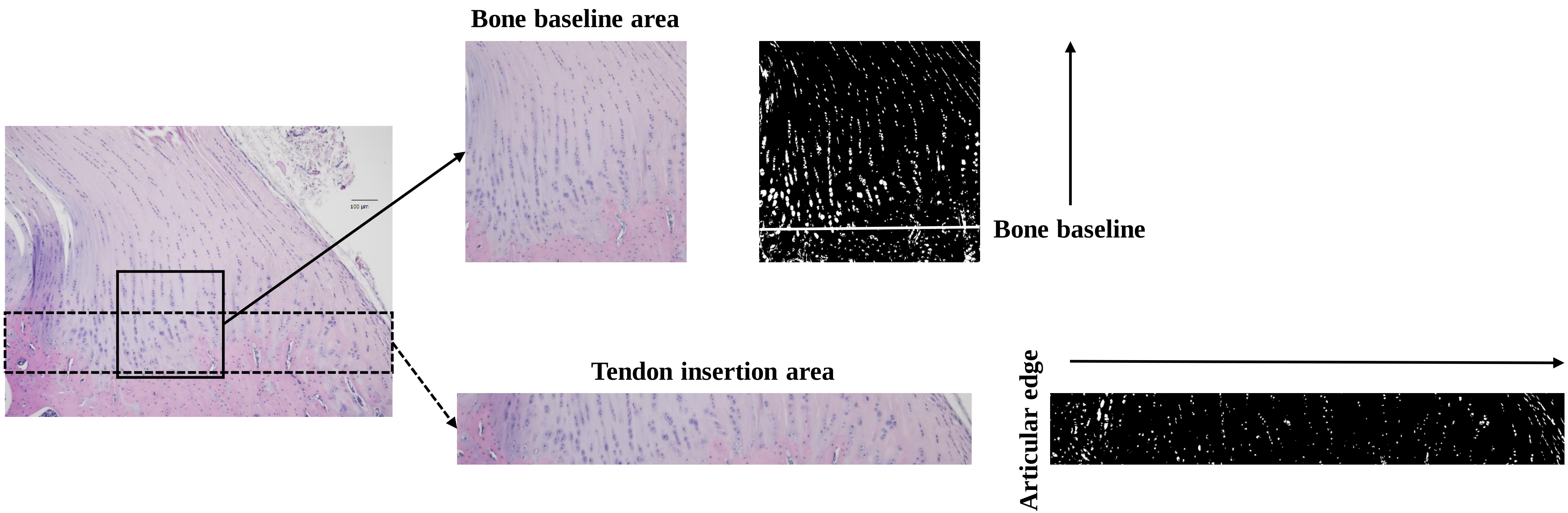

Supplementary, Figure S1

Scatter plot showing the correlation between mechanical properties and BMD. Cross sectional area vs. BMD had significantly positive correlation ($r=0.62$, $p<0.05$).

Supplementary, Figure S2

(a) CT image showing the range of interest for BMD analysis. The image was thresholded at 5 mm distance from the proximal end of the humerus head. Purple rectangle: Range of interest for BMD analysis. Yellow line: The proximal edge of the humerus head. Red line: The lateral edge of the humerus.

(b) CT image showing the axial image for the calculation of cross sectional area. This image was determined at the proximal edge of the humerus head.

Supplementary, Figure S3

Flow diagram for cellular distribution, showing the whole micrographs trimmed into 2 areas (bone baseline area and tendon insertion area), calculated centroid of each cells, sorted by distance from the baseline, and made histogram.
